# A Rare Case of a Dual-Mechanism-Based Thrombocytopenia Secondary to Carbamazepine in an Epileptic Patient

**DOI:** 10.7759/cureus.86674

**Published:** 2025-06-24

**Authors:** Soobia Pathan, Muhammad J Khan, M Khaliq

**Affiliations:** 1 Pharmacology and Therapeutics, Liaquat Institute of Medical and Health Sciences, Thatta, PAK; 2 Acute Medicine, Midland Metropolitan University Hospital, Smethwick, GBR; 3 Pathology, Federal Postgraduate Medical Institute, Lahore, PAK; 4 Pathology, University of Health Sciences, Lahore, PAK

**Keywords:** antiepileptic, carbamazepine, immune-mediated, pathogenesis, thrombocytopenia

## Abstract

Among antiepileptic drugs, carbamazepine often causes various hematological complications, one of which is thrombocytopenia. Reports of immune-mediated and myelosuppressive pathways being involved separately are common, but cases in which both occur together, known as dual-mechanism thrombocytopenia, are not very common. This case described a 35-year-old lady who had epilepsy and presented with unexplained bleeding and a notable decline in the level of her platelets. A high platelet count was found in the peripheral blood, and fewer megakaryocytes were seen in the bone marrow, indicating destruction of platelets in the bloodstream as well as underproduction in the bone marrow. Once carbamazepine was stopped, healthy blood counts came back quickly, indicating a certain connection. It demonstrated that monitoring platelets is important for those on carbamazepine therapy for a long time and that a dual cause should be considered for unexplained or severe low platelet counts.

## Introduction

Carbamazepine, which is an iminostilbene drug, is one of the most widely prescribed antiepileptic medicines and is used to treat focal seizures, generalized tonic-clonic seizures, and trigeminal neuralgia. It mainly prevents the excess opening of voltage-gated sodium channels on neural membranes, reduces their excitability, and prevents repeated firing of neurons [1]. Many people do well with carbamazepine, but it does have several adverse reactions, some of which depend on the dose and others that occur randomly.

Thrombocytopenia is a low platelet count, which is a hematological side effect of radiation therapy. Although not extremely common, it has the potential to be very dangerous [2]. It is clinically noticeable through petechiae, purpura, bleeding in the mouth, or, at times, through life-threatening hemorrhage. Sometimes, thrombocytopenia is caused by the immune-mediated destruction of platelets or by the cessation of the production of platelets by bone marrow megakaryocytes. Drug-induced thrombocytopenia (DIT) is not always noticed because it is reversible, and it occurs when the medication creates antibodies against platelet proteins. Most cases of carbamazepine-induced thrombocytopenia appear to be immune-related; nonetheless, some new studies indicate that in some patients, both mechanisms operate together and are known as dual-mechanism thrombocytopenia [3]. Since people rarely experience this type of epilepsy, it can be difficult to diagnose, as it begins slowly and its symptoms overlap.

Although previous reports have linked thrombocytopenia to carbamazepine, this case shows evidence of both destroyed platelets in the blood and the inability of the bone marrow to produce new ones. This case will increase awareness about this rare problem and will stress the need for prompt observation and stopping the medication to avoid further problems.

## Case presentation

A 35-year-old woman known to have epilepsy visited the emergency department because of noticed petechiae, easy bruising, and bleeding from her gums when she brushed her teeth for five straight days. She mentioned that there had been slightly bloody urine twice, and she also had one episode of bleeding from the nose in the previous 48 hours. Relevant medical records did not mention any recent infections, vaccinations, or newly given drugs. She did not report any symptoms such as fever, joint pain, or weight loss. For several years, her epilepsy was well managed by carbamazepine taken as a single medicine (400 mg twice daily) each day. Her medical history was clear, aside from having epilepsy. No antiplatelets, nonsteroidal anti-inflammatory drugs (NSAIDs), or anticoagulants had been prescribed for her. She claimed to be nonalcoholic and never used illicit drugs. None of the family members were known to have bleeding or autoimmune conditions.

The patient appeared fine and had no changes in vital signs upon checkup. We noticed multiple dark spots without white centers (petechiae) on her forearms and legs and also observed mild bleeding from her gums. The physical examination did not show growth of any organs or lymph nodes. All tests, including neurological and general body functions, were within the standard limits. Results of the first complete blood count showed platelets at 32,000/uL, normal hemoglobin (12.4 g/dL), and a normal white cell count. Microscopic examination found unusually large platelets but no abnormal morphology of red blood cells (RBCs) or white blood cells (WBCs). All coagulation studies (prothrombin time (PT), activated partial thromboplastin time (aPTT)), liver and renal function tests, as well as autoimmune markers (antinuclear antibody (ANA), rheumatoid factor (RF)), fell within the expected values. The panel for viruses (human immunodeficiency virus (HIV), hepatitis B virus (HBV), hepatitis C virus (HCV), cytomegalovirus (CMV), Epstein-Barr virus (EBV)) was all negative.

There was no clear reason found, but since the patient had been taking carbamazepine for a long time, a procedure to look at their bone marrow was performed. This process showed that their marrow cells were normal, but the numbers of megakaryocyte precursors, which help in making blood platelets, were reduced. The use of the Naranjo Adverse Drug Reaction Probability Scale helped in confirming the link between carbamazepine and thrombocytopenia. Many health professionals use this structure of ten questions to assess the risk of an unwanted drug reaction [4]. Here, the calculated score summed to 7, which suggests that carbamazepine is the most probable cause for the patient’s blood problems based on the health professional’s suspicion. Table 1 lists the Naranjo Adverse Drug Reaction Probability Scale for the present case.

**Table 1:** Naranjo Adverse Drug Reaction Probability Scale for the present case ADR: adverse drug reaction; ≥9: definite ADR; 5-8: Probable ADR; 1-4: possible ADR; 0 or less: doubtful ADR Adapted/reproduced from Naranjo et al. [4], with permission

Question	Yes (score)	No (score)	Do not know (score)	Response for the present case	Score
1. Are there previous conclusive reports on this reaction?	+1	0	0	Yes, thrombocytopenia with carbamazepine is reported in the literature	+1
2. Did the adverse event appear after the suspected drug was administered?	+2	-1	0	Yes, the patient developed thrombocytopenia while on carbamazepine	+2
3. Did the adverse reaction improve when the drug was discontinued?	+1	0	0	Yes, platelet counts improved significantly after stopping the drug.	+1
4. Did the adverse reaction reappear upon re-administration of the drug?	+2	-1	0	Not attempted (ethically inappropriate)	0
5. Are there alternative causes that could have caused the reaction?	-1	+2	0	No other cause found (infections, autoimmune, etc., ruled out)	+2
6. Did the reaction reappear with a placebo?	-1	+1	0	Not applicable	0
7. Was the drug detected in the blood (toxic concentrations)?	+1	0	0	Drug level not measured	0
8. Was the reaction more severe when the dose was increased, or less severe when the dose was decreased?	+1	0	0	Dose was stable; no dose modulation observed	0
9. Did the patient have a similar reaction to the same or similar drugs in any previous exposure?	+1	0	0	No previous similar reaction reported	0
10. Was the adverse event confirmed by any objective evidence?	+1	0	0	Yes, confirmed by lab data and bone marrow findings	+1

The cause of dual-mechanism thrombocytopenia (immune-mediated and marrow-suppressive) diagnosed was carbamazepine use. Because of the patient’s drug reaction, we stopped the previous drug and introduced levetiracetam with a dose of 500 mg twice daily. As the patient was not having complications, steroids and intravenous immunoglobulin (IVIG) were not given. Table 2 presents the changes in platelet numbers within the patient’s body.

**Table 2:** Hematological parameters during hospital stay cu.mm: cubic millimeter

Day	Platelet count (per cu.mm)	Smear findings	Notes
Day 1	32,000	Giant platelets present	Baseline
Day 4	46,000	Reduced giant forms	Initial response after cessation
Day 7	75,000	Mild anisopoikilocytosis	Continued upward trend
Day 14	1,22,000	Normal morphology	Full recovery

Platelets were measured each day, and their numbers went up: 46,000/uL on day 3, 75,000/uL on day 7, and 1,22,000/uL by day 14, all while the patient’s condition was improving. The patient was allowed to go home following outpatient care and further neurologic examination.

## Discussion

Over the years of steady carbamazepine use, the patient suffered from severe thrombocytopenia. The evaluation was wide enough to rule out viral infections, conditions related to the immune system, cancer of the blood, or nutrition-based problems as causes for anemia. This significant improvement in platelets after carbamazepine was withdrawn will be very supportive of a DIT diagnosis. Carbamazepine is known to occasionally cause blood-related side effects, although these are uncommon [5]. Bone marrow suppression, by impairing platelet production, and immune-mediated peripheral destruction involving drug-induced antiplatelet antibodies are the two main mechanisms described in the pathogenesis of DIT [6]. Both mechanisms appeared to be occurring simultaneously in this case. While the bone marrow results revealed a reduction in the number of immature megakaryocytes, which indicated decreased platelet generation, the presence of big platelets on the peripheral smear suggested an increased platelet destruction in the bloodstream.

The clinical context summed up to the Naranjo score result of 7, labeling the reaction as "probable," which is aligned with other reports in the literature on antiepileptic drug-caused hematological toxicity [7]. A similar case was published, in which a patient receiving carbamazepine experienced severe thrombocytopenia, but there were no reports on the bone marrow [8]. Our findings showed both damage to the spleen’s outer layer and the bone marrow, which highlighted the necessity of considering dual-pathway injury when there is no clear reason for thrombocytopenia. Several published case reports mentioned carbamazepine-associated thrombocytopenia, and most have explained it as being immune-mediated or marrow-suppressed [9,10]. Dual-mechanism thrombocytopenia, similar to what happened in our case, is quite rare and not widely recognized. This points out that clinicians must watch for possible causes related to blood, especially if their patients are using antiepileptic drugs in the long term. Moreover, our patient experienced thrombocytopenia several years into steady carbamazepine use; therefore, careful monitoring of blood cell levels may help prevent issues in individuals who are at risk [11,12].

This case enriches the small amount of research on dual-pathway carbamazepine-related thrombocytopenia and highlights the need to use a thorough examination as well as a bone marrow test when it is needed. Treatment revolves around rapidly identifying the offending cause and then removing or lowering its amount.

## Conclusions

In this case, carbamazepine caused a notable adverse effect by leading to thrombocytopenia, which involved both platelet destruction by the immune system and suppression of platelet production in the bone marrow. Since the patient recovered quickly after stopping the drug, without any other possible reasons and a Naranjo score of 7, the relationship between the medicine and the adverse event was considered probable. Hematological side effects might occur in patients even many years after receiving stable therapy. Identifying the problem early, testing the marrow when needed, correct evaluation, and removing the offending drug as soon as possible help save patients from severe complications.
